# Quinolone tolerance in *Escherichia coli* due to defects in the adenosine ribonucleotides *de novo* biosynthesis pathway

**DOI:** 10.1002/mlf2.70059

**Published:** 2026-04-16

**Authors:** Weiwei Zhu, Yuejuan Nong, Jie Su, Jingwen Yang, Lina Ma, Yunxin Xue, Dai Wang, Jianjun Niu, Karl Drlica, Xilin Zhao

**Affiliations:** ^1^ State Key Laboratory of Vaccines for Infectious Diseases, Xiang‐An Biomedicine Laboratory, National Innovation Platform for Industry‐Education Integration in Vaccine Research, Department of Laboratory Medicine, School of Public Health Xiamen University Xiamen China; ^2^ Center of Clinical Laboratory, Zhongshan Hospital, School of Medicine Xiamen University Xiamen China; ^3^ Public Health Research Institute and Department of Microbiology, Biochemistry & Molecular Genetics, New Jersey Medical School Rutgers University Newark New Jersey USA

**Keywords:** adenosine ribonucleotides *de novo* biosynthesis, AMP, antibiotic tolerance, PurA/PurB, ROS

## Abstract

Central carbon metabolism is thought to link reactive oxygen species (ROS) with antibiotic‐mediated bacterial death. During enrichment screening of *Escherichia coli* with the first‐generation quinolone oxolinic acid, unstable antibiotic‐tolerant mutants containing deficiencies in *purB* were obtained. Examination of a stable deletion mutant of *purA*, a gene functionally related to *purB*, revealed reduced lethality of oxolinic acid and ciprofloxacin. This deletion mutation had little effect on the minimal inhibitory concentration (MIC) of quinolones, thereby demonstrating that the observed protection from killing was attributable to antibiotic tolerance. AMP synthesis was blocked by the Δ*purA* mutation, and ciprofloxacin tolerance was reversed by exogenous AMP supplementation. Because AMP is a precursor of ATP, interference with ATP synthesis occurs in the Δ*purA* mutant. RNA‐Seq analysis showed that, prior to antibiotic stress, transcript levels of NADH:quinone oxidoreductase genes were reduced by the *purA* deficiency, thereby predisposing *E. coli* to antibiotic tolerance through reduced respiration. During ciprofloxacin exposure, the *purA* deficiency also suppressed the surge in expression of tricarboxylic acid (TCA) cycle and ATP synthesis genes, as well as the accumulation of intracellular ATP and ROS. Thus, wild‐type PurA, and by extension the downstream enzyme PurB, directs AMP toward an antibiotic‐mediated, ROS‐dependent death pathway. Overall, defects in PurA/PurB‐mediated adenosine ribonucleotides *de novo* biosynthesis reveal a novel quinolone tolerance mechanism that is initiated outside central carbon metabolism; tolerance is likely attributable to a limited supply of AMP, resulting in reduced ATP synthesis and suppression of ROS accumulation.

## Impact statement

Previous studies with rapidly growing *Escherichia coli* identified a stress‐mediated death pathway in which defects led to both antibiotic and disinfectant tolerance. In principle, massive disinfectant consumption could lead to tolerance and thereby interfere with antibiotic effectiveness. Since tolerant bacteria cannot be identified by standard susceptibility testing, new clinically implementable methods need to be developed. We report a metabolic pathway, adenosine ribonucleotides *de novo* biosynthesis pathway, likely to serve as a novel biomarker for DNA‐based tolerance surveillance. This study also shows that regulatory networks outside central carbon metabolism can contribute to the lethal metabolic response to antibiotics, thereby opening a new research direction for developing antibiotic adjuvants.

## INTRODUCTION

Although the bacteriostatic action of antibiotics is thought to be sufficient for clinical cure^1^, ^2^, widespread resistance has increased interest in antibiotic lethality^3^, particularly with respect to the lethal metabolic response of cells to antibiotic‐mediated lesions^4^, ^5^, ^6^, ^7^. A central feature of the metabolic response is the production of reactive metabolites. Among these are reactive oxygen species (ROS), which arise as by‐products of respiration and ATP generation^4^, ^5^, ^6^, ^7^, ^8^, ^9^. A study based on screening antibiotic‐tolerant mutations has revealed a broadly shared central death pathway leading from glucose metabolism to cAMP (a death‐signaling molecule) and Crp as a stimulator of the tricarboxylic acid (TCA) cycle^10^. Counteracting this central death pathway is the protective induction of the stringent response and the accumulation of ppGpp (an antideath‐signaling molecule)^11^, ^12^. Moreover, disinfectant tolerance emerged from the same mutations that cause antibiotic tolerance^10^. Since many genes are involved in antibiotic/disinfectant lethality, the emergence of tolerance is likely to be a high‐probability event. Consequently, the widespread use of disinfectants could contribute to the emergence of tolerance. However, the prevalence of tolerance and whether it constitutes a public health problem parallel to resistance remain unknown. To address this issue, facile assays for the detection of tolerance are needed.

Determination of the prevalence of tolerance through microbiological surveys is difficult because of the absence of breakpoints comparable to those used in resistance surveys and the low throughput of time‐consuming kill‐curve assays. Although agar‐based tests are being developed^13^, identification of nucleotide sequence changes associated with tolerance could make DNA‐based test an attractive and robust alternative that could be readily implemented in clinical settings. At present, only a few such nucleotide changes are known, mainly within central carbon metabolism and cellular respiration^10^, ^14^. Because additional metabolic pathways exist that supply substrates for central carbon metabolism, defects in these pathways could interfere with antibiotic‐mediated bacterial killing. Thus, the potential pool of tolerance mutations may be larger than anticipated from central carbon metabolism alone.

One approach for identifying metabolic genes involved in antibiotic lethality is the isolation of tolerant mutants followed by whole‐genome sequencing^10^, ^15^. Such mutants are not killed by an antibiotic, while bacterial growth remains inhibited by the antibiotic. These mutants can be obtained by challenging bacterial cells with antimicrobials whose lethal action requires the accumulation of reactive metabolites^10^. First‐generation quinolones, such as nalidixic acid and oxolinic acid, satisfy this requirement^16^, ^17^, ^18^, ^19^.

In this study, defects in *purA* and *purB* within the adenosine ribonucleotides *de novo* biosynthesis pathway were found to confer *E. coli* tolerance to quinolone‐mediated killing. This pathway, which lies outside central carbon metabolism and respiration, when being defect, mediates quinolone tolerance through inhibition of AMP synthesis and, consequently, downstream ATP synthesis and by‐product ROS production. Through this work, deficiencies in *purA* and *purB* are added to the list of tolerance‐associated mutations that may be used in DNA‐based tolerance surveys. In addition, an approach for expanding the currently known repertoire of bacterial tolerance mutations is identified.

## RESULTS

### Enrichment, identification, and characterization of oxolinic acid‐tolerant mutants

**Figure 1:**
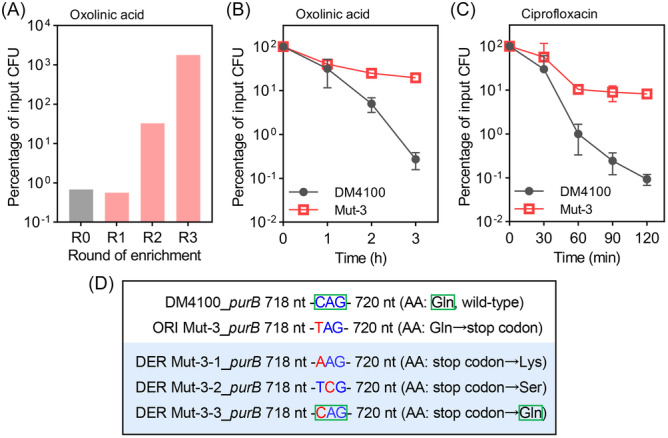
Enrichment, identification, and characterization of oxolinic acid‐tolerant mutants. (A) Percentage of input CFU (colony‐forming unit) after the D4100 parental strain after three rounds of challenge (R1–R3) with 10× MIC oxolinic acid for 3 h. (B, C) Decreased killing of strain Mut‐3 with 10× MIC oxolinic acid (B) and 5× MIC ciprofloxacin (C). Exponentially growing mutant and wild‐type cells were treated with quinolone for the indicated times, and the percentage of input CFU was measured by plating on drug‐free agar and colony counting. For each experiment, at least three biological replicates were performed; error bars represent standard deviations. (D) Identification of the genome alteration sites of the four mutants. The original *purB* mutant was genetically unstable and reverted to either the wild‐type or a mutant allele that corrected the premature stop codon. Green boxes indicate the codon and its encoding amino acid (Gln240) in the wild‐type and revertant (Mut‐3‐3) PurB protein. AA, amino acid; DER mut‐3, mutants producing variations in the *purB* gene derived from strain ORI Mut‐3; ORI mut‐3, original mutant derived from DM4100.

Addition of oxolinic acid at 10× MIC to an exponentially growing culture of *E. coli* resulted in an approximately 100‐fold reduction in the number of viable cells within 3 h (Figure 1A, R0). The quinolone was subsequently removed by washing, and the surviving cells were regrown prior to another round of oxolinic acid treatment. After the second round of enrichment, bacterial survival increased 48‐fold (Figure 1A, R2). After the third round, the total number of bacteria obviously increased (Figure 1A, R3), as expected if resistant mutants within the culture survived oxolinic acid treatment and started proliferation. Cultures obtained from the third round of enrichment were screened to isolate oxolinic acid‐tolerant strains. Resistant mutants, defined as those exhibiting a > 2‐fold increase in MIC compared with the parental (isogenic) susceptible strain^15^, ^20^, were discarded because tolerant mutants are required to exhibit no change in MIC. Among the remaining cells, isolates were obtained that showed an approximately 100‐fold reduction in killing by oxolinic acid with little change in MIC (Figure 1B, Table S1). These isolates also exhibited tolerance to the fluoroquinolone ciprofloxacin, with an approximately 100‐fold increase in survival (Figure 1C, Table S1). One isolate, designated Mut‐3, was selected for further examination.

Whole‐genome sequencing of the Mut‐3 isolate revealed a single non‐synonymous mutation in the coding sequence, consisting of a C‐to‐T substitution at the 718th base position of *purB* (corresponding to amino acid 240). This mutation resulted in premature termination of translation (Figure 1D). Unexpectedly, Mut‐3 exhibited an unstable genotype, as the codon corresponding to amino acid 240 of Mut‐3 PurB frequently reverted from a termination codon (TAG) to the wild‐type *purB* codon (CAG) or other translatable codons (AAG or TCG) after repeated culturing in the absence of antimicrobial agents (Figure 1D).

### Pur‐mediated metabolic pathways

**Figure 2:**
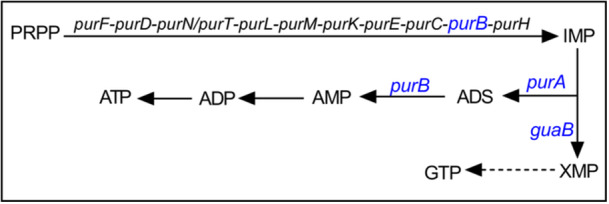
Metabolic pathways involved in PurA and PurB. IMP serves as a substrate for both *purA* and *guaB* in a branched pathway. Dashed lines indicate that intermediate processes have been omitted. ADS, adenylosuccinate; IMP, inosine monophosphate; PRPP, 5‐phosphoribosyl 1‐pyrophosphate; XMP, xanthosine 5‐phosphate. This pathway diagram was created with reference to the KEGG database^22^, ^23^.

**Figure 3:**
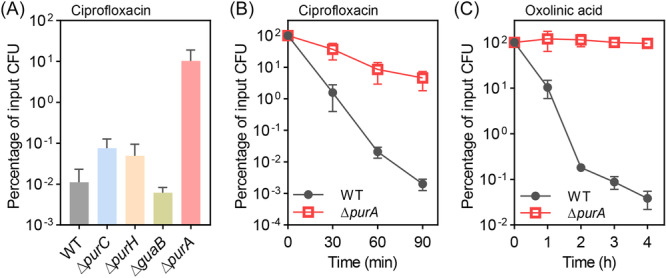
Protective effect of *purA* deficiency for quinolone antibiotics. (A) Percentage of input CFU after wild‐type strain BW25113 and the indicated mutants treated with 5× MIC ciprofloxacin for 120 min. (B, C) Effect of *purA* deficiency on bacterial killing kinetics during treatment with 10× MIC ciprofloxacin (B), or 10× MIC oxolinic acid (C). Exponentially growing *E. coli* cells were treated with antibiotics for the indicated times, and the percentage of input CFU was measured by plating on drug‐free agar and colony counting. At least three biological replicates were performed for each data point; error bars represent standard deviations.

PurB is an adenylate succinate lyase involved in two multi‐gene metabolic pathways in *E. coli*: the conversion of 5‐phospho‐α‐D‐ribose 1‐diphosphate (PRPP) to inosine monophosphate (IMP) and the conversion of IMP to adenosine monophosphate (AMP), which serves as a precursor for ADP and ultimately ATP synthesis (Figure 2)^21^. Deficiency of *purC* or *purH* (Figure 3A), which are located immediately upstream and downstream of *purB*, respectively (as shown in Figure 2), or deficiency of other *pur* genes in the PRPP pathway (*purDEFKLM*) (Figure S1), failed to significantly increase *E. coli* survival (less than a 10‐fold change) during ciprofloxacin treatment at 5× MIC. These data indicated that antibiotic tolerance mediated by *purB* deficiency is not due to the lack of conversion of PRPP to IMP. This observation left the conversion of IMP to AMP as a likely pathway disrupted during the generation of antibiotic tolerance. Since IMP derived from PRPP can also be converted to GTP through the action of *guaB* as part of a branched pathway (Figure 2), a *guaB* deletion mutant was examined for its effect on ciprofloxacin lethality. It was shown that no effect was observed (Figure 3A). Thus, the quinolone tolerance pathway mediated by *purB* deficiency (Figure 2) is distinct from the PRPP‐IMP‐GTP branch. The absence of tolerance following deletion of *guaB* directed attention toward disruption of the IMP‐ATP pathway as a potential explanation for quinolone tolerance.

### *purA* as a tolerance gene

Deletion of *purA*, the first gene in the pathway that, together with *purB*, converts IMP to AMP, resulted in an approximately 1000‐fold increase in *E. coli* survival during ciprofloxacin treatment (Figure 3A,B). As expected, deletion of *purA* protected *E. coli* from being killed by another quinolone antimicrobial (oxolinic acid) (Figure 3C) without affecting the MIC (Table S2). Since plasmid‐mediated overexpression of *purA* reversed the protective effect associated with *purA* deficiency (Figure S2), the mutation was likely responsible for the observed tolerance. Although the absence of *purA* only slightly reduced the rate of exponential growth (a doubling time of 26.0 min for wild‐type *E. coli* compared with 29.5 min for a Δ*purA* mutant), the total yield at stationary phase was decreased by an order of magnitude (Figure S3). PurB also appeared to be critically important for bacterial growth, as indicated by the spontaneous transition of prematurely terminated PurB to a fully translatable protein (isolate Mut‐3, Figure 1D) in the absence of antibiotic exposure. Because a *purB* mutant is unstable and PurB participates in the conversion of both PRPP to IMP and IMP to AMP, whereas PurA participates only in the conversion of IMP to AMP^21^, ^24^ (Figure 2), studies focusing on PurA were expected to be more readily interpreted. Accordingly, a Δ*purA* mutant was used for additional characterization of tolerance.

### *purA* deficiency‐mediated quinolone tolerance is associated with low AMP levels

**Figure 4:**
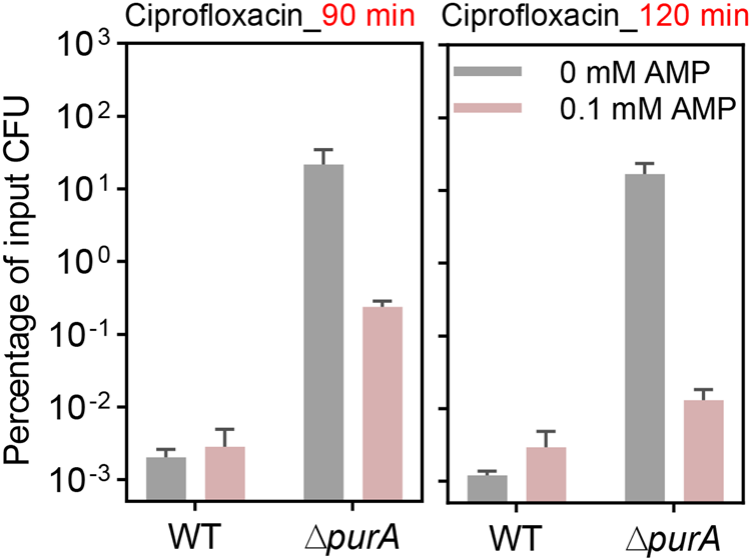
Effect of exogenous AMP on *purA* deficiency‐mediated antibiotic killing. Percentage of input CFU after the wild‐type strain BW25113 and Δ*purA* mutant after 5× MIC ciprofloxacin treatment for 90 min (left) or 120 min (right) following supplementation with AMP. Exponentially growing cells were pretreated with the 0 or 0.1 mM AMP for 15 min and then treated with 5× MIC ciprofloxacin for the indicated times, and the percentage of input CFU was measured by plating on drug‐free agar and colony counting. At least three biological replicates were performed for each experiment; error bars represent standard deviations.

Since previous studies have indicated that low ATP levels are associated with bacterial tolerance and persistence^25^, ^26^, ^27^, ^28^, antibiotic tolerance mediated by *purA* deletion may result from reduced ATP levels due to limitation of the AMP precursor supply. To test this hypothesis, cultures were supplemented with exogenous AMP. At a concentration of 0.1 mM, AMP failed to protect wild‐type cells from ciprofloxacin‐mediated killing; however, survival of the Δ*purA* mutant was reduced by 10‐100‐fold after 90 min of ciprofloxacin treatment and by 100‐1000‐fold after 120 min (Figure 4). At 0.1 mM, AMP had no effect on the ciprofloxacin MIC (Table S3). The observation that exogenous AMP overcame ciprofloxacin tolerance indicates that defects in *purA* mediate antibiotic tolerance by limiting intracellular AMP levels.

### *purA* deficiency limits cellular respiration and ROS accumulation

**Figure 5:**
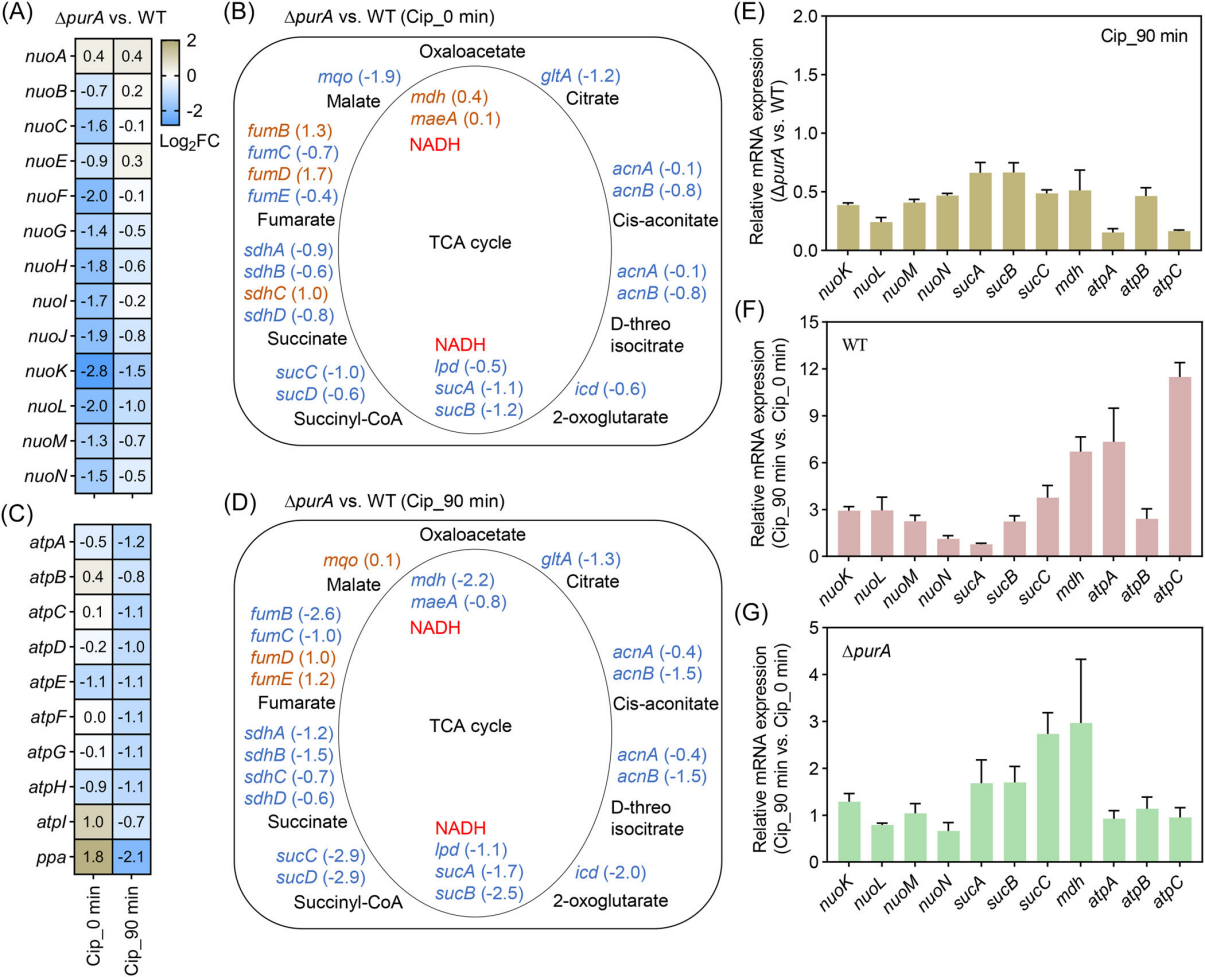
*purA* deficiency affects transcript levels of the tricarboxylic acid (TCA) cycle and oxidative phosphorylation genes. (A–D) RNA‐Seq analysis was performed to determine the relative transcript levels of the TCA cycle and oxidative phosphorylation genes in exponentially growing Δ*purA* mutant and wild‐type strain (BW25113) treated with 5× MIC ciprofloxacin for 0 or 90 min. (A, C) Relative transcript levels of NADH:quinone oxidoreductase (A) and ATP synthase genes (C) in the Δ*purA* mutant compared to the wild‐type strain. FC, fold change. (B, D) Relative transcript levels of TCA cycle genes for 0 min (B) and 90 min (D). Metabolites in the TCA cycle are shown in black font. Orange font denotes genes with upregulated expression, while blue font denotes genes with downregulated expression. The Arabic numerals in parentheses represent the log₂‐transformed values of the differences in gene transcript levels between the Δ*purA* mutant and the wild‐type strain. Metabolic processes that produce NADH are labeled NADH in red font. (E–G) RT‐qPCR analysis of the relative transcript levels of the indicated genes involved in the TCA cycle and oxidative phosphorylation after 5× MIC ciprofloxacin treatment for 0 or 90 min. At least three biological replicates were performed for each experiment; error bars represent SEM.

As a precursor for ATP synthesis, low AMP levels could limit cellular respiratory processes that produce ATP. To characterize these effects at the transcriptional level, RNA‐Seq analysis was performed. In the absence of ciprofloxacin, deletion of *purA* reduced transcript levels of NADH:quinone oxidoreductase genes (Figure 5A), suggesting that *purA* deficiency decreases the ability of *E. coli* to metabolize NADH and inhibits early steps in cellular respiration. The TCA cycle and ATP synthesis were then examined, as these processes produce NADH and utilize the energy released from NADH, respectively. The *purA* deficiency reduced transcript levels of TCA cycle genes. Among metabolic processes that generate NADH, decreased expression of *lpd‐sucA‐sucB* was observed, whereas a slight increase in expression of *mdh‐maeA* was detected (Figure 5B). Expression patterns of genes related to ATP synthesis were variable following *purA* deletion (e.g., the transcript level of *atpE* was reduced 1.1‐fold, whereas that of *ppa* (encoding inorganic pyrophosphatase) was increased 1.8‐fold) (Figure 5C). These expression patterns made changes in ATP levels difficult to predict from gene expression data alone. Thus, in the absence of antibiotic stress, the primary metabolic node affected by *purA* deficiency appears to be the downregulation of NADH:quinone oxidoreductase.

Antibiotic stress has been expected to stimulate bacterial central carbon metabolism and cellular respiration^4^, ^5^, ^6^, ^7^. When *E. coli* was treated with ciprofloxacin for 90 min, transcript levels of TCA‐cycle genes were reduced by *purA* deficiency, particularly those of *lpd‐sucA‐sucB* and *mdh‐maeA* (Figure 5D). Transcript levels of genes related to ATP synthesis were also reduced by *purA* deficiency (Figure 5A,C). Subsequent RT‐qPCR analysis confirmed the RNA‐Seq results. For example, under ciprofloxacin stress, lower expression levels of *nuoKLMN* (NADH:quinone oxidoreductase genes), *sucABC* and *mdh* (TCA cycle genes), and *atpABC* (ATP synthase genes) were observed in the Δ*purA* mutant compared with the wild‐type strain (Figure 5E). This phenotype was attributed to ciprofloxacin treatment stimulating high‐level expression of these genes in the wild‐type strain (Figure 5F), whereas the *purA* defect restricted this surge in expression (Figure 5G).

**Figure 6:**
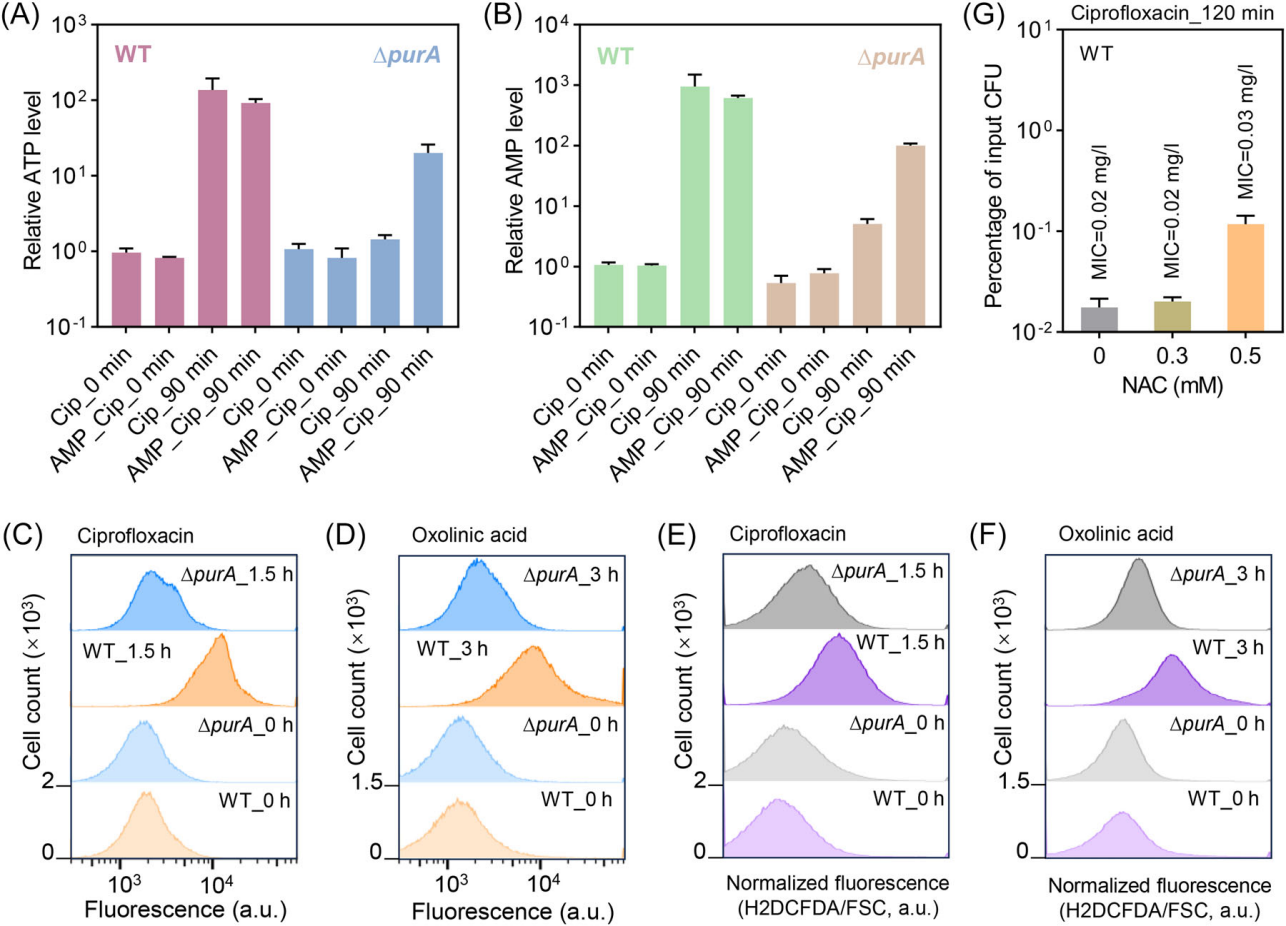
*purA* deficiency suppresses ATP and reactive oxygen species (ROS) levels of *E. coli* during antibiotic treatment. (A, B) Deficiency in *purA* restricts the increase in cellular ATP (A) and AMP (B) stimulated by ciprofloxacin. Wild‐type BW25113 and Δ*purA* mutant cells were treated with 5× MIC ciprofloxacin for 0 or 90 min. Relative ATP and AMP levels were respectively calculated based on wild‐type intracellular ATP and AMP levels in the absence of AMP and without ciprofloxacin treatment. AMP in the horizontal coordinate indicates that 0.1 mM AMP was added for co‐incubation 15 min before ciprofloxacin treatment. Data are averages of three biological replicates; error bars represent standard deviations. (C, D) *purA* deficiency lowers quinolone‐stimulated intracellular ROS levels. Exponentially growing wild‐type (BW25113) and Δ*purA* mutant cells were pretreated with the ROS dye (carboxy‐H2DCFDA) for 20 min and then treated with 10× MIC ciprofloxacin (C) or with 10× MIC oxolinic acid (D) for the indicated times. (E, F) Normalized fluorescence for panels C and D according to *E. coli* cell size, calculated as carboxy‐H2DCFDA fluorescence divided by the Forward Scatter‐Area (FSC‐A) value. Samples were subjected to flow cytometry for analysis of intracellular ROS levels. Similar results were obtained in three replicate experiments. a.u., arbitrary unit. (G) Percentage of input CFU after the wild‐type strain BW25113 after 5× MIC ciprofloxacin treatment following supplementation with exogenous N‐acetylcysteine (NAC). Exponentially growing cells were pretreated with the indicated NAC for 15 min and then treated with 5× MIC ciprofloxacin for 120 min, and the percentage of input CFU was measured by plating on drug‐free agar and colony counting. At least three biological replicates were performed for each experiment; error bars represent standard deviations. The MIC of ciprofloxacin with the wild‐type strain after the addition of the indicated concentrations of NAC are indicated.

ATP levels tend to correlate positively with cellular respiration^7^, ^29^. Determination of intracellular ATP levels in *E. coli* revealed that, in the absence of antibiotic stress, ATP levels in the Δ*purA* mutant were not significantly different from those in the wild‐type strain (Figure 6A). Notably, the surge in ATP levels during ciprofloxacin stress was limited by *purA* defects (Figure 6A), consistent with suppression of the transcript levels of *atp* and *ppa* during ciprofloxacin treatment of the Δ*purA* mutant (Figure 5C). Addition of exogenous AMP eliminated the *purA*‐deficiency‐mediated limitation of the ATP surge during ciprofloxacin stress (Figure 6A). Trends in intracellular AMP levels before and after ciprofloxacin stress, as well as in the presence or absence of exogenous AMP, were aligned with those observed for ATP levels (Figure 6B). These data corroborate the observation that AMP reverses *purA*‐deficiency‐mediated ciprofloxacin tolerance (Figure 4).

Restricted ATP synthesis is expected to reduce ROS levels because ROS are by‐products of cellular respiration^7^, ^9^, ^10^. Intracellular ROS levels in the Δ*purA* mutant and wild‐type cells were characterized using the fluorescent dye carboxy‐H2DCFDA^30^. In the absence of quinolone‐mediated stress, the positions of peak ROS fluorescence were identical for mutant and wild‐type cells (Figure 6C,D; bottom two columns). After treatment with ciprofloxacin (1.5 h) or oxolinic acid (3 h), the fluorescence peaks of wild‐type cells shifted farther to the right (indicating increased ROS levels) than those of the Δ*purA* mutant (Figure 6C,D; top two columns). Since quinolone stress causes bacterial cells to elongate^31^, which might interfere with single‐cell fluorescence analysis, carboxy‐H2DCFDA fluorescence was normalized according to cell size. After normalization, ciprofloxacin‐ and oxolinic acid‐mediated stress was found to induce a rightward shift in the ROS fluorescence peak, whereas *purA* defects blocked this shift (Figure 6E,F). Addition of the antioxidant N‐acetylcysteine at 0.5 mM slightly increased the MIC of ciprofloxacin against *E. coli* (from 0.02 mg/l to 0.03 mg/l), but *E. coli* survival was enhanced by 6.7‐fold (Figure 6G). This result is consistent with previous findings showing that the addition of an ROS scavenger (dimethyl sulfoxide) reduces ciprofloxacin lethality and that catalase/peroxidase (KatE‐KatG) deficiency increases ciprofloxacin lethality^10^. It is therefore concluded that *purA* deficiency suppresses the surge in intracellular ROS associated with quinolone stress, thereby explaining the observed tolerance phenotype.

## DISCUSSION

In the present work, oxolinic acid was used as a probe to screen *E. coli* mutants for quinolone tolerance. Defects in *purA* and *purB* were identified as novel markers of tolerance. We identified a metabolic pathway, adenosine ribonucleotides *de novo* biosynthesis, in which defects counteract the lethal cellular response to quinolone‐mediated stress that would otherwise lead to ROS accumulation and bacterial death.

### Blocking AMP synthesis underlies *pur* deficiency‐mediated quinolone tolerance

Direct evidence supporting the conclusion that tolerance arises from low AMP levels is provided by the reversal of *purA*‐deficiency‐mediated tolerance through supplementation with exogenous AMP (Figure 4), the product of PurA/PurB. Involvement of low AMP levels in *purA*‐based tolerance was linked to central carbon metabolism through the association of *purA* deficiency with reduced ATP synthesis and decreased ROS accumulation (Figures 5 and 6). Previous studies have indicated that low ATP levels are associated with bacterial tolerance and persistence^25^, ^26^, ^27^, ^28^, which is consistent with the present findings (Figure 6A). It is emphasized that attenuation of ATP synthesis *per se*, rather than a reduction in ATP levels alone, may be responsible for suppression of quinolone killing, because such attenuated synthesis would be accompanied by reduced ROS accumulation.

AMP carries two negative charges. Although it cannot cross the cell membrane of *E. coli* directly^32^, specific uptake pores for AMP do exist^33^. Measurement of intracellular AMP in *E. coli* revealed that pre‐incubation with AMP had no effect on intracellular AMP levels in the Δ*purA* mutant and wild‐type cells in the absence of ciprofloxacin, whereas in the presence of ciprofloxacin, exogenous AMP obviously increased intracellular AMP levels in the Δ*purA* mutant but not in the wild‐type strain (Figure 6B). Ciprofloxacin stress triggers a marked elevation in intracellular ATP and its precursor AMP levels in *E. coli*. When *purA* deficiency impairs AMP biosynthesis, two mechanisms may account for the contribution of exogenous AMP to intracellular AMP levels in the mutant under antibiotic stress. First, a defect in endogenous AMP synthesis may stimulate the activity of AMP‐specific uptake pores. Second, ciprofloxacin‐induced ROS increase membrane permeability, thereby promoting AMP diffusion. In the Δ*purA* mutant, the large transmembrane AMP gradient drives AMP uptake following ciprofloxacin‐mediated membrane permeabilization. By contrast, ciprofloxacin stimulates robust endogenous AMP production in wild‐type cells, thereby diminishing the transmembrane gradient of AMP and thus reducing gradient‐driven diffusion‐based AMP uptake; consequently, exogenous AMP has little effect on intracellular AMP levels.

The behavior observed for quinolones is not observed for all antibiotics under all conditions. For example, in a previous study, *purA* defects were shown to increase the lethal action of colistin and aminoglycosides in *E. coli* ^34^, a result that contrasts with the decreased lethality observed for quinolones in the present study. The increased lethality of colistin in a *purA*‐deficient mutant was attributed to membrane hyperpolarization resulting from suppression of ATP synthesis, which itself is a consequence of reduced AMP synthesis^34^. The opposite effect of *purA* deficiency on these two antibiotic groups (quinolones and polymyxins) can be explained by differences in their lethal mechanisms: quinolones kill largely through the accumulation of toxic metabolites (ROS)^4^, ^8^, ^35^, whereas polymyxins kill primarily through membrane damage, which is ROS‐independent^36^. Accordingly, these two groups of compounds have been categorized as strongly (quinolones) and weakly (polymyxins) metabolism‐dependent antibiotics^37^. In another example, aminoglycoside lethality may depend on ROS^4^ or may be ROS‐independent^36^, ^37^, depending on experimental conditions. Such differences in the behavior of antimicrobial compounds can facilitate the formulation of highly lethal antibiotic combinations^36^, ^37^.

It is noted that, in the absence of antibiotics, differences in intracellular ATP levels between the *purA* mutant and the wild‐type strain appear to vary with cell density. For example, in a previous study, cultures grown to OD_600_ = 0.8 exhibited intracellular ATP levels that were significantly lower in the *purA* mutant than in wild‐type cells. The difference was smaller at OD_600_ = 0.5^34^, but under the experimental conditions in the present study (OD_600_ = 0.25–0.3), no obvious difference in intracellular ATP levels was observed between the *purA* mutant and wild‐type cells (Figure 6A). These observations suggest that ATP synthesis can be maintained at levels comparable to those of wild‐type cells in the *purA* mutant during the low cell density of early exponential‐phase growth. However, as cell density and metabolic activity increase during the mid‐to‐late exponential phase, the limited supply of AMP in the *purA* mutant reduces ATP synthesis even in the absence of antimicrobial exposure.

### Genetic instability of *purB* is a potential factor in antibiotic treatment failure

AMP synthesis from the adenosine ribonucleotides *de novo* biosynthesis pathway appears to be genetically regulated during lethal stress. Quinolone treatment enriched a *purB* mutant that tolerates quinolone killing. After the removal of quinolone‐mediated stress, a single‐base alteration in a *purB* premature‐stop mutant was observed to spontaneously revert *purB* to the wild‐type sequence or to other translatable PurB variants (Figure 1D) which outcompete the premature‐stop mutant. This makes the *purB* mutant culture genetically unstable. Such genetic instability of *purB* may derive from PurB functioning in both IMP synthesis and AMP/GMP synthesis. A deficiency in both IMP and AMP/GMP synthesis, although still allowing bacterial growth in rich medium, would starve bacterial cells of purine nucleotide needed for DNA replication. A reversion single‐base mutation, regardless through a spontaneous DNA replication error, a replication fork‐stoppage induced SOS response, or stress‐induced mutation, as long as it restores PurB function, would confer the revertant growth advantage, gradually outcompete the *purB* mutant, and eventually dominate the entire bacterial population following normal (no external stress exposure) growth and passages.

Such a conclusion is further supported by the inability to obtain a *purB* knockout mutant^38^. Genetic instability involving other genes has also been reported in the context of heteroresistance^39^. This genetic form of stress response can complicate antibiotic susceptibility determination: in the presence of antibiotics, genes may mutate to tolerate or resist drug stress; however, when antibiotics are withdrawn, the genes may revert to the wild‐type state, resulting in improved bacterial growth.

In addition to mediating quinolone tolerance, purine synthesis defects may also promote the evolution of bacterial resistance. Defects in the purine synthesis pathway create specific metabolic bottlenecks, decreasing *E. coli* growth rates and energy production. This, in turn, diminishes the bactericidal effects of antibiotics, promotes the formation of persisters, and provides a window for resistance mutations to develop^40^. In methicillin‐resistant *Staphylococcus aureus* (MRSA), mutations that block purine synthesis or transport are often linked to increased levels of c‐di‐AMP, a key mechanism by which they enhance resistance to β‐lactam antibiotics^41^.

**Figure 7:**
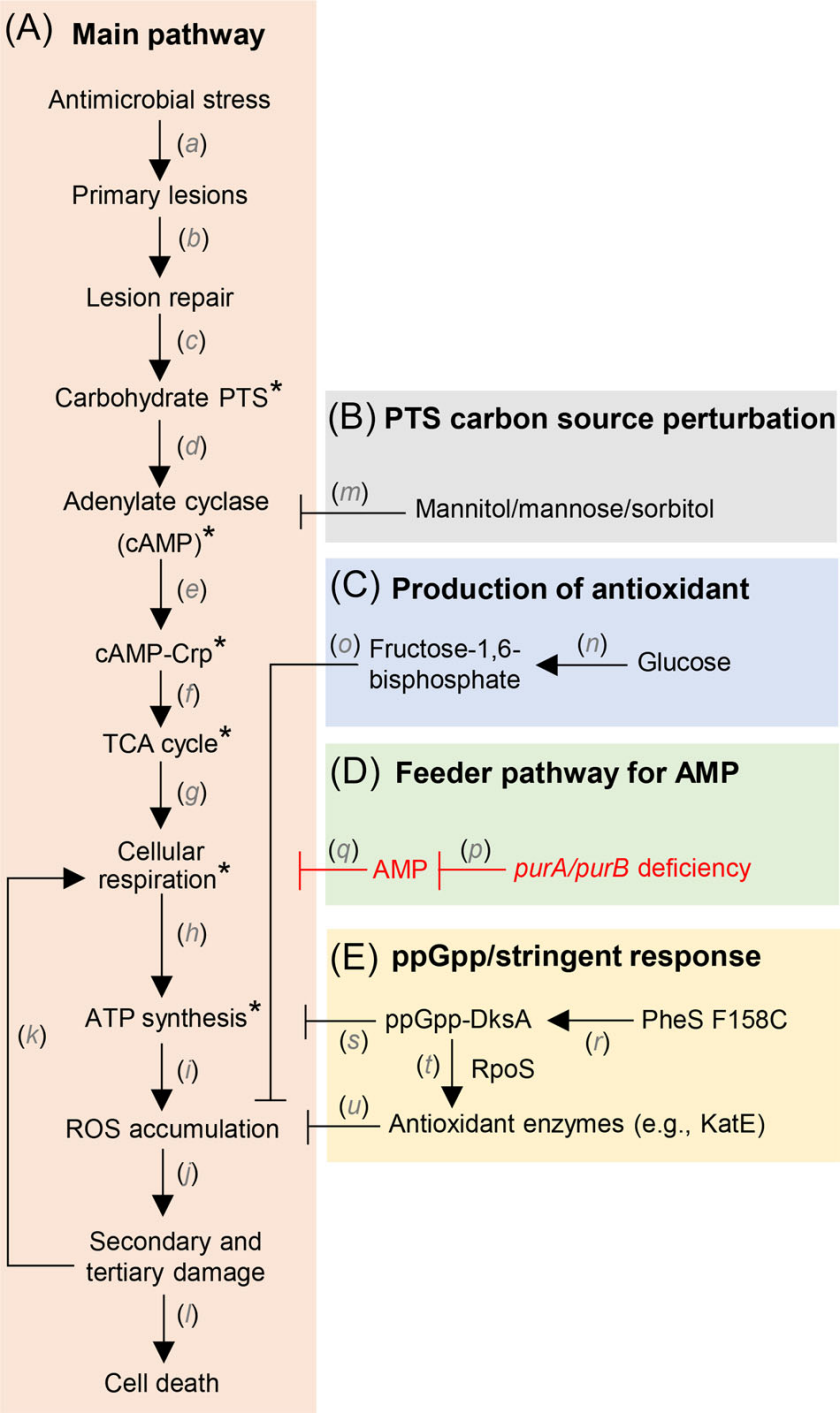
The mechanisms underlying antibiotic tolerance mediated by alterations in metabolic pathways in rapidly growing *E. coli* cells. (A) Main pathway to antimicrobial‐mediated bacterial death^4^, ^7^, ^10^. Primary lesions at specific targets by bactericidal antibiotics (*a*) stimulate lesion repair (*b*), which increases demand for energy, and leads to activation of the phosphotransferase system (PTS)‐cAMP‐Crp cascade (*c–e*), which involves glucose uptake. A surge in central carbon metabolism (*f*) occurs that produces NADH required for cellular respiration (*g*) and the generation of ATP (*h*). Accumulation of metabolic by‐products (ROS) (*i*) leads to oxidative damage of macromolecules (*j*), further increasing the demand for ATP (*k* and *h*) and the additional accumulation of ROS (*i*). Oxidative damage (*h*–*k*) results in cell death (*l*). Asterisks (*) indicate where mutations disrupt the pathway and cause antibiotic tolerance. (B) PTS carbon source perturbation^45^. Exogenous mannitol, mannose, or sorbitol reduces antibiotic lethality by restricting cAMP‐Crp‐mediated regulation (*m*) and inhibiting downstream processes (*e–l*). (C) Production of antioxidant^46^. Fructose‐1,6‐bisphosphate generated from exogenous glucose (*n*) acts as an antioxidant (*o*) during antibiotic‐mediated cell death (*l*). (D) Feeder pathway for AMP synthesis (present work). Outside the main pathway, disruption of AMP synthesis (*p*) by *purA*/*purB* deficiency in the adenosine ribonucleotides *de novo* biosynthesis pathway results in restriction of cellular respiration (*q*), ultimately inhibiting antibiotic‐mediated cell death (*l*). (E) ppGpp/stringent response^42^. A defect in PheS tRNA synthetase creates disinfectant tolerance by inducing the stringent response (*r*), which inhibits ATP synthesis (*s*) and stimulates expression of RpoS‐dependent antioxidant enzymes (*t* and *u*).

In summary, as presented schematically in Figure 7A, a death pathway is stimulated by antibiotics and disinfectants when these agents are added to rapidly growing cultures of *E. coli*. The demand for repair of lesions generated in bacterial cells stimulates carbohydrate uptake through the phosphotransferase system (PTS), followed by activation of the cAMP‐Crp cascade. This process promotes the TCA cycle, cellular respiration, ATP synthesis, ROS accumulation, and ultimately cell death^10^. This pathway is defined by mutations and treatments that block killing without affecting the MIC. Some perturbations pre‐induce protection from ROS. For example, a defect in phenylalanine aminoacyl‐tRNA synthetase has been shown to promote broad‐spectrum tolerance to disinfectants even in the absence of disinfectant stress^42^. In the present work, a *purA* defect suppresses AMP levels and consequently ATP levels, which are expected to reduce the formation of cAMP. As a result, cAMP‐Crp‐mediated positive regulation of genes involved in respiration and the TCA cycle would be reduced. Such a scenario would require downstream suppression of ATP synthesis to influence upstream synthesis of cAMP through a feedback loop. Numerous ancillary pathways likely exist that are not depicted in Figure 7, as indicated by tolerance mutations identified under growth‐defect conditions and by cross‐antibiotic tolerance resulting from mutations in non‐antibiotic stress systems^15^, ^20^, ^43^, ^44^. It is expected that identification of a full repertoire of tolerance mutations will provide the biomarkers necessary for establishing a DNA‐based molecular diagnostic method for surveillance of antibiotic and disinfectant tolerance.

## MATERIALS AND METHODS

### Bacterial strains, culture, and reagents

*E. coli* strains used in this study are listed in Table S4. The accuracy of mutations in strains recovered from the Keio collection was verified by nucleotide sequence determination after PCR amplification using primers listed in Table S5. All bacteria were cultured in Luria‐Bertani (LB) medium at 37°C with shaking at 200 rpm. Ciprofloxacin and oxolinic acid were purchased from Sigma‐Aldrich Corp. Adenosine monophosphate (AMP) was purchased from Sangon Biotech Inc., and carboxy‐H2DCFDA was obtained from Thermo Fisher Scientific.

### Antibiotic susceptibility determination

MIC was determined by a twofold broth dilution method^10^. Exponentially growing cultures were diluted to 10^5 ^cells/ml, and AMP was added as needed, followed by antimicrobial addition and incubation at 37°C for 16 h. MIC was defined as the lowest antibiotic concentration at which no turbidity development could be observed visually.

### Bacterial killing assays

Overnight bacterial cultures were diluted 100‐fold and regrown to mid‐exponential phase (OD_600_ = 0.25–0.3). AMP was added 15 min before antimicrobials. Aliquots were taken at various times and serially diluted 10‐fold in 0.9% saline; then diluted samples (10 μl) were spotted in triplicate on LB agar followed by incubation for 24 h. The percentage of input CFU (colony‐forming unit) was determined by visual CFU relative to an untreated culture sampled at the time of antimicrobial addition.

### Enrichment of tolerant mutants

To obtain oxolinic acid‐tolerant mutants, cultures of *E. coli* strain DM4100 were grown to OD_600_ = 0.25–0.3 in LB medium at 37°C with shaking at 200 rpm, and then treated with 10× MIC oxolinic acid for 3 h. Oxolinic acid reduced the survival of *E. coli* by 100‐ to 1000‐fold. *E. coli* cells were concentrated by centrifugation (4°C, 4600*g*, 5 min) and resuspended in fresh LB medium. Two more centrifugation and culture resuspension steps were performed to remove residual oxolinic acid. Cultures were then diluted 100‐fold and incubated overnight for an additional round of enrichment. Following three rounds of enrichment, the cultures were washed and plated on oxolinic acid‐free agar; resulting colonies were tested for growth on agar containing 3× MIC of oxolinic acid. Bacteria that grew only on agar plates lacking oxolinic acid were used for subsequent MIC determinations and time‐kill experiments. Bacteria with no significant change in drug MIC and high‐level survival to oxolinic acid treatment were considered oxolinic acid‐tolerant mutants.

### Whole‐genome sequencing

Chromosomal DNA was extracted from overnight cultures of both wild‐type and mutant *E. coli* strains using a bacterial genomic DNA isolation kit (Tiangen Biotech Co.), following the manufacturer's protocol. Whole‐genome sequencing was performed by Novogene Co., Ltd., with subsequent comparative genomic analysis against the *E. coli* BW25113 reference genome (GenBank Accession Number: CP009273.1). The identity of mutations was validated by PCR amplification and subsequent DNA sequencing.

### Plasmid construction and allelic exchange

Chromosomal DNA from strain BW25113 was used as a template to amplify the *purA* gene and its promoter. After digestion of DNA with restriction endonucleases *Bam*HI and *Sal*I, the *purA* gene was inserted into the pACYC184 vector to obtain p*purA*. Enzyme digestion and PCR‐amplified *purA* fragment sequencing were used to verify correct insertion into the plasmid. A *guaB* deletion mutant was created by homologous recombination using a one‐step chromosome gene inactivation method^47^. pCP20 plasmid was used to eliminate antibiotic resistance marker genes when desired^47^.

### Measurement of cellular ATP and AMP levels

Exponentially growing (OD_600_ = 0.25–0.3) cultures of the *purA* mutant and wild‐type strain BW25113 were treated with 5× MIC ciprofloxacin for 90 min, followed by centrifugation (12,000*g*, 2 min) to concentrate the cells. After washing twice with cold 0.9% saline, the cell pellets were resuspended in the lysis buffer provided in the assay kit (below). Using a VC‐750 ultrasonic processor (Sonics & Materials), the cells were lysed by repeated (10‐sec treatment followed by 10‐sec rest) ultrasonic treatment on ice at 40% power for 2 min. Post‐lysis centrifugation (12,000*g*, 2 min) removed cell debris. The supernatant fluid was used for the determination of ATP and AMP levels using ATP Assay Kit (Beyotime) and AMP Assay Kit (colorimetric) (Abcam) according to the vendor's protocol. Luminescence and absorbance were measured in 96‐well plates with Infinite E Plex (TECAN). The ATP and AMP concentrations were calculated and normalized to cell number.

### Measurement of ROS levels

A final concentration of 10 μM Carboxy‐H2DCFDA^6^ was added to cultures to detect total intracellular ROS. Exponentially growing (OD_600_ = 0.25–0.3) cultures of the *purA* mutant and wild‐type strain BW25113 were treated with 10× MIC ciprofloxacin for 1.5 h or 10 × MIC oxolinic acid for 3 h. Aliquots taken at various times were washed twice with pre‐chilled 1 ml phosphate‐buffered saline and then analyzed by flow cytometry (CytoFLEX; Beckman Coulter Inc.). For all test samples, 10^5^ cells were recorded under the 525/40 nm band pass filter (FITC channel), and the generated FCS file was used for FlowJo‐X analysis. Fluorescent signal intensity was normalized to cell size to rule out cellular filamentation effects triggered by quinolone treatment. Normalization was carried out by dividing carboxy‐H2DCFDA fluorescence by the Forward Scatter‐Area (FSC‐A) value (in flow cytometry, FSC‐A is positively correlated with cell size).

### RNA‐Seq analysis

Exponentially growing (OD_600_ = 0.25–0.3) cultures of the Δ*purA* mutant and strain BW25113 were treated with 5× MIC ciprofloxacin for 0 or 90 min. Sample cells were collected by centrifugation (4°C, 4600*g*, 10 min), frozen with liquid nitrogen, and then shipped to Novogene for RNA extraction, library construction, and RNA‐Seq analysis. An RNA extraction kit (Tiangen Biotech) was used to prepare total bacterial RNA. Following the manufacturer's protocol, sequencing libraries were constructed with the NEBNext Ultra Directional RNA Library Prep Kit for Illumina (New England Biolabs) and assigned unique index codes to distinguish each sample. To enrich mRNA, ribosomal RNAs were removed with the Ribo‐Zero Kit (Illumina). Subsequently, the enriched mRNA was fragmented in NEBNext RNA First Strand Synthesis Reaction Buffer (New England Biolabs). mRNA was reverse‐transcribed into cDNA using random hexamer primers, followed by double‐stranded cDNA synthesis. The double‐stranded cDNAs were subjected to end repair, poly‐A tailing, and sequencing adapter ligation. Fragment size selection (150–200 bp) was carried out with the AMPure XP system (Beckman Coulter), followed by PCR amplification. The final library was purified using the AMPure XP system^48^. Sequencing was performed on Illumina HiSeq. 2500/MiSeq instruments. Read counts per gene were generated using HTSeq (version 0.6.1)^49^, ^50^. RNA‐Seq data processing was conducted with the DEGSeq package (v1.20.0)^51^, ^52^, ^53^, with results deposited in the Gene Expression Omnibus (GEO) database (Accession No: GSE292923).

### Total RNA extraction and cDNA synthesis

For RT‐qPCR analysis, exponentially growing (OD_600_ = 0.25–0.3) cultures of BW25113 and Δ*purA* mutant strains were treated with 5× MIC ciprofloxacin for 0 or 90 min. Samples were centrifuged (12,000*g*, 4°C, 2 min) to collect cells. Subsequently, total bacterial RNA was extracted using an RNA extraction kit (TransGen Biotech) according to the vendor's technical manual. Total bacterial RNA was reverse‐transcribed with random primers, and single‐stranded cDNAs were synthesized according to the technical manual of the All‐in‐One 5× RT MasterMix kit (Applied Biological Materials). The resulting cDNA samples were stored at −80°C.

### RT‐qPCR

2× Universal SYBR Green Fast qPCR Mix (Abclonal) was utilized for amplification of targeted PCR products using primers listed in Table S5 with a qTOWER3 G instrument (Analytik Jena AG). The thermal cycling conditions were as follows: initial denaturation at 95°C for 3 min, then 40 cycles at 95°C for 5 s followed by 60°C for 30 s. The fluorescence value of 16S rRNA was used as an internal standard to calculate the relative expression of targeted genes using the 2^−ΔΔCt^ method^54^.

### Statistical analyses

All experiments had at least three biological replicates except for RNA‐Seq analysis. Each data point represents the mean of replicate experiments; error bars represent standard deviations unless otherwise stated.

## AUTHOR CONTRIBUTIONS

**Weiwei Zhu**: Conceptualization; data curation; formal analysis; investigation; methodology; visualization; writing—original draft; writing—review and editing. **Yuejuan Nong**: Data curation; formal analysis; investigation; software; validation; visualization; writing—original draft. **Jie Su**: Data curation; investigation; methodology; software; visualization. **Jingwen Yang**: Investigation; methodology. **Lina Ma**: Investigation; methodology. **Yunxin Xue**: Resources. **Dai Wang**: Resources. **Jianjun Niu**: Conceptualization; formal analysis; resources; writing—original draft; writing—review and editing. **Karl Drlica**: Conceptualization; formal analysis; visualization; writing—original draft; writing—review and editing. **Xilin Zhao**: Conceptualization; funding acquisition; project administration; resources; supervision; writing—original draft; writing—review and editing.

## ETHICS STATEMENT

No animal or human subjects were involved in this study.

## CONFLICT OF INTERESTS

The authors declare no conflict of interests.

## Supporting information

Figure S1. Effect of inosine‐5'‐phosphate biosynthesis pathway defects on ciprofloxacin lethality.
Figure S2. Plasmid‐expressed purA reversed purA deficiency‐mediated ciprofloxacin tolerance.
Figure S3. Effect of purA deficiency on the growth of *E. coli*.
Table S1. MIC of oxolinic acid and ciprofloxacin against *E. coli* strains DM4100 and Mut‐3.
Table S2. MIC of various antibiotics against *E. coli* strains BW25113 and ΔpurA.
Table S3. MIC of ciprofloxacin against wild‐type BW25113 and mutant strains.
Table S4. Bacterial strains and plasmids used in the study. Table S5. Primers used in the study.

## Data Availability

Data supporting the findings of this study are all shown in the main text and supplementary materials. The RNA‐Seq data are available from the GEO database (Accession No: GSE292923).
