# Supplementary material for: Quinolone tolerance in Escherichia coli due to defects in the adenosine ribonucleotides de novo biosynthesis pathway
Source: mLife. 2026 Apr 16;5(2):217–28. doi: 10.1002/mlf2.70059 (PMC13131326; doi:10.1002/mlf2.70059)
Supplement: Supplementary file 1 — Figure S1. Effect of inosine‐5'‐phosphate biosynthesis pathway defects on ciprofloxacin lethality. Figure S2. Plasmid‐expressed purA reversed purA deficiency‐mediated ciprofloxacin tolerance. Figure S3. Effect of purA deficiency on the growth of E. coli. Table S1. MIC of oxolinic acid and ciprofloxacin against E. coli strains DM4100 and Mut‐3. Table S2. MIC of various antibiotics against E. coli strains BW25113 and ΔpurA. Table S3. MIC of ciprofloxacin against wild‐type BW25113 and mutant strains. Table S4. Bacterial strains and plasmids used in the study. Table S5. Primers used in the study. [file MLF2-5-217-s001.pdf]

Supplementary Materials

Quinolone tolerance in *Escherichia coli* due to defects in the adenosine ribonucleotides *de novo* biosynthesis pathway

Weiwei Zhu[1,#], Yuejuan Nong[1,#], Jie Su[1], Jingwen Yang[1], Lina Ma[1], Yunxin Xue[1], Dai Wang[1], Jianjun Niu[2,*], Karl Drlica[3], Xilin Zhao[1,*]

[1] State Key Laboratory of Vaccines for Infectious Diseases, Xiang-An Biomedicine Laboratory, National Innovation Platform for Industry-Education Integration in Vaccine Research, Department of Laboratory Medicine, School of Public Health, Xiamen University, Xiamen, China.

[2] Center of Clinical Laboratory, Zhongshan Hospital, School of Medicine, Xiamen University, Xiamen, China.

[3] Public Health Research Institute and Department of Microbiology, Biochemistry & Molecular Genetics, New Jersey Medical School, Rutgers University, Newark, New Jersey, USA.

[#] Weiwei Zhu and Yuejuan Nong contributed equally to the work.

* Address correspondence to: zhaox5@xmu.edu.cn, and niujianjun@xmu.edu.com.

Table of Contents

Supplementary Figures

Figure S1. Effect of inosine-5'-phosphate biosynthesis pathway defects on ciprofloxacin lethality.

Figure S2. Plasmid-expressed *purA* reversed *purA* deficiency-mediated ciprofloxacin tolerance.

Figure S3. Effect of *purA* deficiency on growth of *E. coli*.

Supplementary Tables

Table S1. MIC of oxolinic acid and ciprofloxacin against *E. coli* strains DM4100 and Mut-3.

Table S2. MIC of various antibiotics against *E. coli* strains BW25113 and Δ*purA*.

Table S3. MIC of ciprofloxacin against wild-type BW25113 and mutant strains.

Table S4. Bacterial strains and plasmids used in the study.

Table S5. Primers used in the study.

Supplementary References

Supplementary Figures

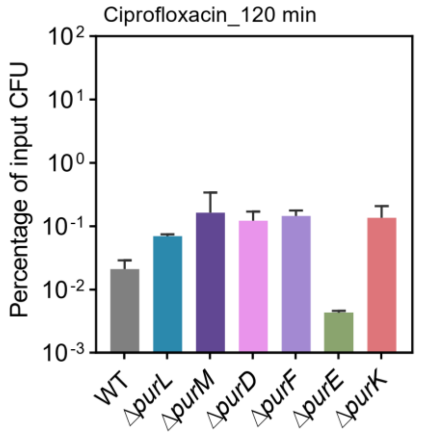

Figure S1. Effect of inosine-5'-phosphate biosynthesis pathway defects on ciprofloxacin lethality. Exponentially growing *E. coli* cells were treated with 5× MIC ciprofloxacin for 120 min for both wild-type (strain BW25113) and indicated mutants. The percentage of input CFU was determined by plating on drug-free agar and colony counting. At least three biological replicate were performed; error bars represent standard deviations.

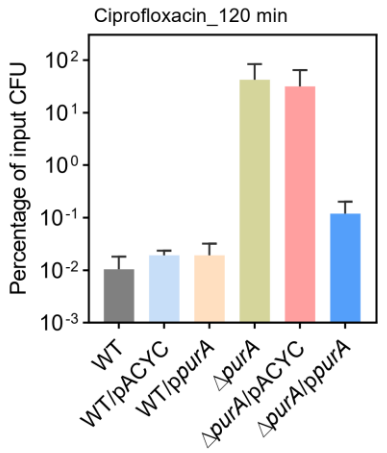

Figure S2. Plasmid-expressed *purA* reversed *purA* deficiency-mediated ciprofloxacin tolerance. Exponentially growing *E. coli* cells were treated with 5× MIC ciprofloxacin for 120 min. Wild-type strain, BW25113; pACYC is the plasmid vector; p*purA* is a *purA*-containing plasmid. The percentage of input CFU was determined by plating on drug-free agar and colony counting. At least three biological replicates were performed; error bars represent standard deviations. pACYC represents pACYC184 plasmid; p*purA* represents pACYC184 carrying *purA*.

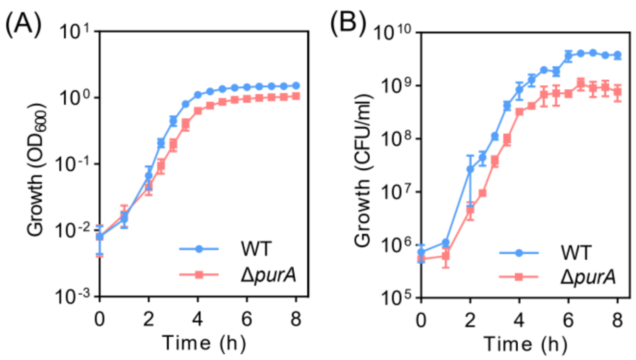

Figure S3. Effect of *purA* deficiency on growth of *E. coli*. Overnight bacterial cultures were diluted 2000-fold at time zero and re-grown in LB medium. The optical density (**A**) and colony-forming unit (CFU) (**B**) of the cultures were measured at various time points, and then the relationship curve between time and OD_{600} value or CFU was plotted. At least three biological replicates were performed and error bars represent standard deviations.

Supplementary Tables

Table S1. MIC of oxolinic acid and ciprofloxacin against *E. coli* strains DM4100 and Mut-3.

Chemicals	Strain/MIC (mg/l)	
	DM4100	Mut-3
Oxolinic acid	0.375	0.5
Ciprofloxacin	0.018	0.018

Table S2. MIC of various antibiotics against *E. coli* strains BW25113 and Δ*purA*.

Chemicals	Strain/MIC (mg/l)	
	BW25113	Δ*purA*
Oxolinic acid	0.6	0.6
Ciprofloxacin	0.02	0.02
kanamycin	4	4
Meropenem	0.05	0.05

Table S3. MIC of ciprofloxacin against wild-type BW25113 and mutant strains.

Strain	BW25113	Δ*purH*	Δ*purC*	Δ*purD*	Δ*purL*	Δ*purF*
MIC (mg/l)	0.02	0.02	0.02	0.02	0.02	0.02
Strain	Δ*purM*	Δ*purE*	Δ*purK*	Δ*guaB*	BW25113+0.1 mM AMP	Δ*purA*+0.1 mM AMP
MIC (mg/l)	0.02	0.02	0.02	0.02	0.02	0.02
Strain	BW25113/pACYC		BW25113/p*purA*	Δ*purA*/pACYC		Δ*purA*/p*purA*
MIC (mg/l)	0.02		0.02	0.02		0.02

Table S4. Bacterial strains and plasmids used in the study.

No.	Strain	genotype	Source/reference
26	DM4100	*E. coli* W3110 *cysB242*(Am)	(1)
27	Mut-3	DM4100 *purB* (C718T), by enrichment with oxolinic acido	This work
60	BW25113	*E. coli* wild-type *rrnB*3 Δ*lacZ*4787 *hsdR*514 Δ(*araBAD*)567 Δ(*rhaBAD*)568 *rph-1*	(2)
2131	Δ*purA*	BW25113 Δ*purA*::kan^R, Keio Collection, JW4135	(2)
2132	Δ*purC*	BW25113 Δ*purC*::kan^R, Keio Collection, JW2461	(2)
2133	Δ*purD*	BW25113 Δ*purD*::kan^R, Keio Collection, JW3969	(2)
2134	Δ*purE*	BW25113 Δ*purE*::kan^R, Keio Collection, JW0512	(2)
2135	Δ*purF*	BW25113 Δ*purF*::kan^R, Keio Collection, JW2309	(2)
2136	Δ*purH*	BW25113 Δ*purH*::kan^R, Keio Collection, JW3970	(2)
2137	Δ*purK*	BW25113 Δ*purK*::kan^R, Keio Collection, JW0511	(2)
1483	Δ*purL*	BW25113 Δ*purL*::kan^R, Keio Collection, JW2541	(2)
2138	Δ*purM*	BW25113 Δ*purM*::kan^R, Keio Collection, JW2484	(2)
1484	Δ*guaB*	BW25113 deletion of the *guaB* gene	This work
1016	BW25113/pACYC	BW25113 contains pACYC184 empty vector	This work
1486	BW25113/p*purA*	BW25113 contains the pACYC184 recombinant vector carrying the *purA* gene	This work
1487	Δ*purA*/pACYC	Δ*purA* contains pACYC184 empty vector	This work
1488	Δ*purA*/p*purA*	Δ*purA* contains the pACYC184 recombinant vector carrying the *purA* gene	This work
**Plasmids**		**genotype**	**Source/reference**
pCP20		rep_{pSC101}^{ts}*bla cat cI*857P_R	(3)
pACYC184		rep_{p15A} *bla* Cm^R Tc^R	(4)
pKD3		rep_{R6K} *bla* FRT *cat* FRT	(5)
pKD46		rep_{Psc101}^{ts} *bla* P_{araBAD} γβ exo	(5)
p*purA*		The *purA* gene was inserted into the pACYC184 vector.	This work

Table S5. Primers used in the study.

Primer Name	Sequence（5′--3′）	Usage
F-*purB*-check	AAGTGCAGGCGAAAGTTC	Upstream primer for *purB* mutant identification
R-*purB*-check	CAACGCATTGTCTTCAACA	Downstream primer for *purB* mutant identification
R-KO-check[a]	GCTTGCTGTCCATAAAACCG	Universal downstream primer for KO mutant identification
F-*purA*-KO-check	CTACATGTTGAGGAAAACGA	Upstream primer for Δ*purA* identification
F-*purC*-KO-check	CACACCCAGGAGTGATAAAG	Upstream primer for Δ*purC* identification
F-*purD*-KO-check	GCGCCACTTCCGCCATTAAT	Upstream primer for Δ*purD* identification
F-*purE*-KO-check	GAGTTGTGCACCACAGGAGT	Upstream primer for Δ*purE* identification
F-*purF*-KO-check	GTAAGTGCTCTGAGATGTGG	Upstream primer for Δ*purF* identification
F-*purH*-KO-check	GCTAACGCTCTCTGTAATAG	Upstream primer for Δ*purH* identification
F-*purK*-KO-check	CCGACGAAGTGCTGGAAAAC	Upstream primer for Δ*purK* identification

Continued Table S5.		
F-*purL*-KO-check	TGCGCCAGATGGCAACTTAT	Upstream primer for Δ*purL* identification
F-*purM*-KO-check	CTGTTAGAATTGCGCCGAAT	Upstream primer for Δ*purM* identification
F-*guaB*-del	GCCCATGCTACGTATCGCTAAAGA AGCTCTGACGTTTGACGTGTAGGC TGGAGCTGCTTC	To delete the *guaB* gene from chromosome
R-*guaB*-del	GAAGAGAATCAGGAGCCCAGACG GTAGTTCGGGGACTCTTCCATATG AATATCCTCCTTAG	
F-*guaB*-check	GATAGTAACCCGCCCTTCGG	Upstream primer for Δ*guaB* identification
R-*guaB*-check	GCCCACAGTTCGCAGTAAAC	Downstream primer for Δ*guaB* identification
F-*purA*-pACYC	CGGGATCCCAGACTGATCGAGGTC ATT	Upstream primer for *purA* insertion into pACYC184
R-*purA*-pACYC	ACGCGTCGACTGTGTCGGATAGCG	Downstream primer for *purA* insertion into pACYC184
F-pACYC-check	ACTATGGCGTGCTGCTAG	Upstream primer for p*purA* identification
R-pACYC-check	ACTGGGTTGAAGGCTCTC	Downstream primer for p*purA* identification
F-16S-RT	CTTACGACCAGGGCTACACAC	For RT-qPCR
R-16S-RT	CGGACTACGACGCACTTTATG	For RT-qPCR
F-*nuoK*-RT	GGTCTGGTTATCCGTCGCAA	For RT-qPCR
R-*nuoK*-RT	AAGGCCGATACTCGCTTCTG	For RT-qPCR
F-*nuoL*-RT	CACTGCGGGCTTCTTCAGTA	For RT-qPCR
R-*nuoL*-RT	GGCGTGAGCGTGAATTTGTT	For RT-qPCR
F-*nuoM*-RT	CGTCTGATCGCCTACACCTC	For RT-qPCR
R-*nuoM*-RT	GTCGCGGGTATGGATACGTT	For RT-qPCR
F-*nuoN*-RT	CCTGATGTCCAGCCCGTATC	For RT-qPCR
R-*nuoN*-RT	CCGCCAGCACGTAGAACTTA	For RT-qPCR
F-*sucA*-RT	CTTCATCGACCTGGTGTGCT	For RT-qPCR
R-*sucA*-RT	GGTTAACCATCTCGGTGGCA	For RT-qPCR
F-*sucB*-RT	GGTGTGTTCGGTTCCCTGAT	For RT-qPCR
R-*sucB*-RT	TTCGCGACCATCGATCAGAC	For RT-qPCR
F-*sucC*-RT	ACCGGCTAACTTCCTTGACG	For RT-qPCR
R-*sucC*-RT	AACACCCACTTCTGCTACCG	For RT-qPCR
F-*mdh*-RT	GACCAAACGCATCCAGAACG	For RT-qPCR
R-*mdh*-RT	GTACTGACCGTCGCCTTCAA	For RT-qPCR
F-*atpA*-RT	TATCGTGAACTGGCAGCGTT	For RT-qPCR
R-*atpA*-RT	CCAGGTAACCACGTTCTGCT	For RT-qPCR
F-*atpB*-RT	CGTTCAATCACTGGGCGTTC	For RT-qPCR
R-*atpB*-RT	CGGCACATTCAGGATCCACT	For RT-qPCR
F-*atpC*-RT	AAACAGCACGGTCACGAAGA	For RT-qPCR
R-*atpC*-RT	GCCGTGAGAGCTGCTAATGT	For RT-qPCR

a: R-KO-check is a universal downstream primer paired with F-genes-KO-check, designed to be inside the kanamycin resistance gene. F: forward; R: reverse

Supplementary References

1. Sternglanz R, DiNardo S, Voelkel KA, Nishimura Y, Hirota Y, Becherer K, Zumstein L, Wang JC. 1981. Mutations in the gene coding for *Escherichia coli* DNA topoisomerase I affect transcription and transposition. Proc Natl Acad Sci U S A 78:2747-51.
2. Baba T, Ara T, Hasegawa M, Takai Y, Okumura Y, Baba M, Datsenko KA, Tomita M, Wanner BL, Mori H. 2006. Construction of *Escherichia coli* K-12 in-frame, single-gene knockout mutants: the Keio collection. Mol Syst Biol 2:2006.0008.
3. Cherepanov PP, Wackernagel W. 1995. Gene disruption in *Escherichia coli*: TcR and KmR cassettes with the option of Flp-catalyzed excision of the antibiotic-resistance determinant. Gene 158:9-14.
4. Chang AC, Cohen SN. 1978. Construction and characterization of amplifiable multicopy DNA cloning vehicles derived from the P15A cryptic miniplasmid. J Bacteriol 134:1141-56.
5. Datsenko KA, Wanner BL. 2000. One-step inactivation of chromosomal genes in *Escherichia coli* K-12 using PCR products. Proc Natl Acad Sci U S A 97:6640-5.
